# Flashed Müller-Lyer and Poggendorff Virtual Illusions

**DOI:** 10.1177/20416695211015699

**Published:** 2021-05-26

**Authors:** Stuart Anstis, Patrick Cavanagh

**Affiliations:** Department of Psychology, University of California, San Diego, La Jolla, United States; Department of Psychology, Glendon College, CVR York University, Toronto, ON, Canada Department of Psychological and Brain Sciences, Dartmouth College, Hanover, NH, USA

**Keywords:** motion perception, flash grab, Poggendorff, Muller-Lyer, illusion

## Abstract

A moving frame can dramatically displace the perceived location of stimuli flashed before and after the motion. Here, we use a moving frame to rearrange flashed elements into the form of classic illusions. Without the moving frame, the initial arrangement of the flashed elements has no illusory effect. The question is whether the frame-induced displacement of position precedes or follows the processes underlying the illusions. This illusory offset of flashed chevrons does generate a Müller-Lyer illusion and the illusory offset of two line segments does create a Poggendorff illusion. We conclude that the site where the frame-induced position shift emerges must precede the site at which the Müller-Lyer and Poggendorf illusions arise.

Where do we see objects? The perceived position of flashed targets does not depend entirely on their physical location but is also strongly influenced by their context, in particular by the presence of moving frames that can drag flashed objects along with them (Cavanagh & Anstis, 2013; Özkan et al., 2021; Whitney, 2002; Whitney & Cavanagh, 2000). Thus, in Movie 1, a vertical bar flashes repetitively, always in the same position. A moving textured rectangle fades in, and this perceptually drags the bar to left and right.

**Movie 1.** The moving background rectangle perceptually drags the fixed-position flashing bar along with it.

## Müller-Lyer Illusion

Movie 2 starts with two pairs of superimposed chevrons flashing at the same locations, one above the other. The chevrons alternate over time pointing alternately left and right. They are in counterphase so that when the upper chevron points left and the lower chevron points right vice versa.

**Figure 1.**
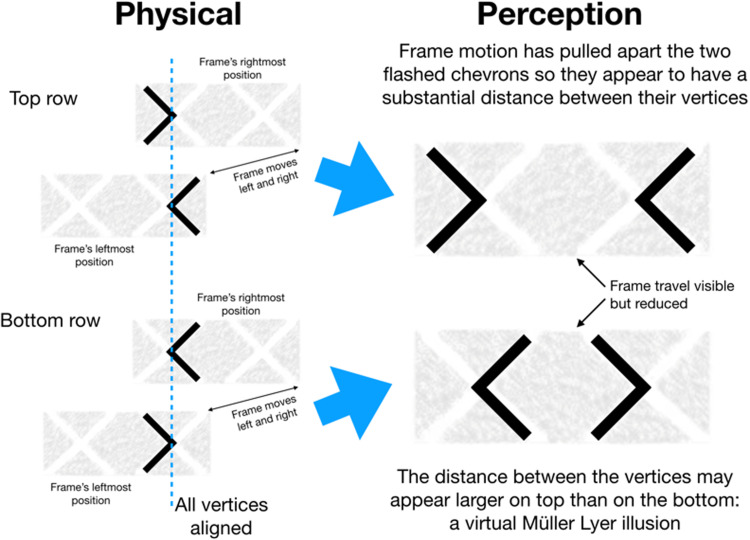
*Left:* The physical arrangement of the moving displays. *Right:* Typical perceived locations. The moving frames pull the superimposed flashed chevrons apart. The upper, outward-pointing chevrons look further apart than the lower, inward-pointing chevrons. This is a virtual Müller-Lyer illusion.

After a few seconds, a textured rectangular frame containing two large white X’s fades in at each location, moving repetitively to left and right. These moving frames grab the flashed chevrons, subjectively dragging them apart. The chevrons are still superimposed on the screen, but they appear to be separated by about the distance through which the rectangles move (Figure 1). Notice that the upper pair of chevrons appear to point outwards and the lower pair of chevrons appear to point inwards. As a result, the space between the two upper chevrons may look larger than between the lower pair (the actual separation is always zero). So the frame effect appears to separate out the chevrons enabling a virtual Müller-Lyer illusion that makes the upper gap look larger than the lower.

**Movie 2.** The chevron pairs at top and bottom are superimposed and flash in place. In the absence of the moving background, there is no apparent separation between them, let alone a difference in the separation between the top pair and that between the bottom pair. When it appears, the moving textured background perceptually drags these chevrons apart. The upper chevrons point outwards and the lower ones inwards. This now produces a virtual Müller-Lyer illusion—the upper pair look further apart than the lower pair.

## Poggendorff Illusion

In Movie 3, two vertically separated oblique lines flash in alternation. A moving tall outline rectangle then fades in, moving left and right at 1.3 Hz. The moving frame effect makes the two flashing lines appear to lie on either side of the moving rectangle: the upper line on the right and the lower line to the left (Figure 2). For many observers, although not all, they appear approximately collinear. This is a control condition that demonstrates the effect of the moving frame on the flash positions and allows us to adjust the vertical positions of the two lines so that they appear collinear for most observers. Because the outline rectangle leaves the lines visible in the center, it does not itself generate a Poggendorff illusion.

**Movie 3.** Two vertically separated oblique lines flash in alternation. An outline rectangle fades in. This puts the lines subjectively on either side of the rectangle and (for some observers) the two lines appear to line up. The rectangle fades out, confirming the vertical separation between the flashed lines. A solid black rectangle fades in. The same two lines now look misaligned—the lower line looks too low, owing to the Poggendorff illusion.

When the outline rectangle fades out in Movie 3, we see again that the two oblique lines are vertically separated. A solid, black vertical rectangle then fades in, with the same dimensions as the outline rectangle. The two lines again appear to lie on either side of the rectangle, but now for many observers, the lines that originally looked collinear with the outline rectangle now appear misaligned with the left line, such that if it were extended it would come out below the right-hand line. This is a virtual Poggendorff illusion. Although the two lines and the rectangle do not form a classic Poggendorff figure on the screen, they are perceptually rearranged by the frames motion to create a Poggendorff figure that supports an illusion of offset.

## Conclusion

What is the order of perceptual processes? We suggest that the moving frames shift the flashed chevrons (Müller-Lyer) or oblique lines (Poggendorff) into new perceived locations. These new subjective layouts then resemble well-known geometrical illusions **(**Movies 2 and 3**)** which induce additional shifts to the flashed components. We conclude that the processes underlying the illusions must follow the frame-induced processes that create the shift. In other words, the elements of a geometrical illusion do not need to be in the appropriate configuration on the screen or on the retina; they only need to look as though they are. This is somewhat counter intuitive. One might expect that position would be seen first and motion second, perhaps as a later perceptual *add-on*. But Walls (1942) knew better. He described motion as *the most ancient and primitive* form of vision. As he put it, “To the animals which invented the vertebrate eye, and held the patents on most of the features of the human model, the visual registration of *movement* was of the greatest importance” (Walls, 1942, p. 342).

## Supplementary Material

Supplementary material

Supplementary material

Supplementary material
